# Radiographic Assessment of Pediatric Condylar Fractures after Conservative Treatment with Functional Appliances—A Systematic Review

**DOI:** 10.3390/ijerph17249204

**Published:** 2020-12-09

**Authors:** Edoardo Staderini, Romeo Patini, Michele Tepedino, Giulio Gasparini, Maria Antonietta Zimbalatti, Francesca Marradi, Patrizia Gallenzi

**Affiliations:** 1 Postgraduate School of Orthodontics, Fondazione Policlinico Universitario A. Gemelli IRCCS, Università Cattolica del Sacro Cuore, Largo Francesco Vito 1, 00168 Rome, Italy; edoardo.staderini@unicatt.it (E.S.); mazimbalatti@hotmail.it (M.A.Z.); f.marradi@gmail.com (F.M.); patrizia.gallenzi@unicatt.it (P.G.); 2 Department of Biotechnological and Applied Clinical Sciences, University of L’Aquila, Viale S.Salvatore, Edificio Delta 6, 67100 L’Aquila, Italy; 3 Maxillofacial Surgery Unit, Fondazione Policlinico Universitario A. Gemelli IRCSS, Università Cattolica del Sacro Cuore, Largo Francesco Vito 1, 00168 Rome, Italy; giulio.gasparini@policlinicogemelli.it

**Keywords:** condylar remodeling, bone remodeling, radiography, panoramic, cone-beam computed tomography, condylar fracture, pediatric patients, children, conservative treatment, functional appliances, growth and development

## Abstract

Background: To evaluate the effectiveness of conservative treatment with functional appliances for condylar fractures in pediatric age. Methods: Four electronic databases (PubMed, EBSCO, Scopus, and Web of Science) were consulted with no restriction of publication status or year, up to 31 August 2020. Selection criteria: based on the PICOS criteria, the selection criteria were set for observational human studies, with at least 10 patients and six months of follow-up. The study population included pediatric patients (aged 5–16 years), with unilateral or bilateral condylar fracture, treated with functional appliances. Condylar remodeling and mandibular growth were analyzed through sequential radiographic examinations. Data collection and analysis: Two independent reviewers carried out title-abstract screening, and a senior investigator was involved to solve any disagreement. The quality of the evidence was assessed through the Canada Institute of Health Economics (IHE) quality appraisal checklist, and the National Institutes of Health (NIH) quality assessment tool. Results: A total of 971 articles were retrieved from the electronic search; among them, three studies met the eligibility criteria. A moderate risk of bias was detected in all the studies, due to common limitations (absence of multicenter studies, prospective design, blindness of the investigators, patients’ drop-out). At follow-up examinations (between 6 months and 4.9 years), the difference of condylar neck length between the “injured” and “healthy” side was approximately 2 mm, while the anteroposterior condylar width discrepancy was recorded up to 1 mm. Conclusions: Short- and long-term data revealed that conservative treatment with functional appliances led to partial or full radiological recovery of the joint morphology, along with good to excellent functional results. Patients’ age has a crucial role on the treatment choice, and the type of fracture (presence of condylar displacement, or dislocation) is also a major prognostic indicator of the radiologic outcome. Limitation: To confirm the effectiveness of functional appliances, more prospective clinical long-term follow-up studies with homogeneous samples of condylar fractures are deemed necessary. Registration: The study protocol was registered on PROSPERO (CRD42020205650).

## 1. Introduction

Condylar fractures are the most common maxillofacial trauma in children and adolescents (32–56% of all facial fractures). [1,2] When a traumatic injury occurs in pediatric age, mandibular growth and condylar remodeling can lead to a gradual return of proper joint function, occlusion, and facial symmetry. Through endochondral ossification, the mandibular skeletal unit (bone and cartilage) grows upward and backward to maintain the rami and condyles in contact with the skull base [3]. The treatment strategy depends on the patient’s age and type of fracture (level, presence of displacement/dislocation). [4]

Orthopedic treatment with functional appliances is one of the conservative approaches indicated for isolated intracapsular condylar fractures in growing age (<16 years), [4] as it is a relatively safe treatment (no surgical complications) [5] and it is more advantageous than intermaxillary fixation (poor oral hygiene, and impaired nutrition, respiration due to absence of mouth opening) [6,7]. It involves the combined use of functional appliances, soft diet, analgesic drugs, and myofunctional therapy [8].

The orthopedic treatment of condylar fractures in pediatric age performs roles of:

- reducing muscle activity of the anterior temporals muscle in the short-term (≤3 months) [9], and balancing the muscle activity on both sides [10]. Even if scientific evidence is missing, it has been hypothesized that occlusal splints act as an adjunct to relieve pain and tenderness of the masticatory muscles [11,12]. This may be due to the fact that orthopedic appliances induce elongation of the masseter muscles (“muscular stretching”) [13], as well as a transitory reduction of jaw-closing muscle activity (“muscular relaxation”) [14,15].
- improving function: in combination with joint mobilization exercises, orthopedic appliances avoid any restriction of mandibular movements due to postoperative pain, muscle protective co-contraction, surgical scars, and adherences [16]; based on the theory of craniofacial growth proposed by Moss et al., a “scar-free technique” can preserve the integrity of the functional matrix, so that the genetic guidance will direct the soft tissue envelope of the temporomandibular joint to rebuild the condylar process [17].
- avoiding occlusal instability due to inadequate mandibular vertical growth of the mandible, e.g., compensatory teeth overeruption on the “healthy” side [18].

Although conservative treatment with functional appliances has already been performed for many years, very few long-term reports are available regarding its effectiveness; therefore, the use of functional appliances seems to be more justified by reduced risks than better outcomes [5]. Many studies have compared the treatment outcomes of condylar fractures t sequential radiographic examinations (panoramic radiographies, cone-beam computed topographies) [8,19,20]. Vertical changes of mandibular growth can be quantified with lengthening of the ramus on the affected side(s), while condylar remodeling can be expressed by morphologic changes of the condyle (size and shape) [21,22]. In unilateral condylar fractures, the difference between the “healthy” and the “injured” sides is the gold standard parameter [2,23]; in bilateral condylar fractures, a homogeneous control group of non-treated patients is needed [8,24].

The objective of this study was to assess radiologically the mandibular growth and condylar remodeling in children with condylar fractures after conservative treatment with functional appliances.

The present study was reported according to Preferred Reporting Items for Systematic Reviews and Meta-Analyses (PRISMA) guidelines (Supplementary material Table S1) [25].

## 2. Materials and Methods

### 2.1. Search Strategy

The study protocol was registered on PROSPERO, the international prospective register of systematic reviews (CRD42020205650).

Based on the “conceptual approach”, a preliminary literature screening was performed to identify synonyms and variants of each search term and to optimize the research question [26]. The electronic search was carried out on four online databases (Medline, Scopus, Web of Science, EBSCO). A manual search was further conducted on the reference list of the included papers from full-text screening and their reference lists.

On 31 August 2020, the electronic search was performed to include all the articles, without restrictions on year or publication status.

The combination of keywords was based on PICOS (P: population; I: intervention; C: comparison; O: outcome; S: study) criteria:

- population: (((((((((Mandibular Fractures * (All Fields)) OR Craniocerebral trauma * (All Fields)) OR Jaw diseases * (All Fields)) OR Jaw * (All Fields)) OR Dental Occlusion, Traumatic * (All Fields)))
- intervention: (((((((((Jaw Fixation Techniques * (All Fields)) OR Diet * (All Fields)) OR diet therapy * (All Fields)) OR Pharmacologic Actions * (All Fields)) OR Occlusal Splints * (All Fields)) OR Orthodontics * (All Fields)) OR Dental Health Services * (All Fields)) OR Myofunctional Therapy* [All Fields]) OR Conservative treatment * (All Fields)))
- comparison: (((child * (All Fields)) OR Adult Children * (All Fields)) OR adolescent * (All Fields)))
- outcome: (((((((((((((Facial Asymmetry * (All Fields)) OR Fracture Healing * (All Fields)) OR Temporomandibular ankylosis * (All Fields)) OR Temporomandibular Joint Disorders * (All Fields)) OR (Growth and development * (All Fields))) OR Dental Occlusion * (All Fields)) OR Jaw Relation Record * (All Fields)) OR Mouth Rehabilitation * (All Fields)) OR Pain Measurement * (All Fields)) OR Quality of Life * (All Fields)) OR treatment outcomes * (All Fields)) OR evaluation * (All Fields)) OR Malocclusion * (All Fields)))
- studies: ((((Observational Study (Publication Type)) OR Clinical Trial (Publication Type)) OR epidemiologic studies) OR Case Reports (Publication Type))

Supplementary material (Table S2) provides the complete search strategy.

### 2.2. Study Selection

The eligibility criteria were the following:

- Age: 5–16 years;
- Type of fracture: unilateral or bilateral condylar fracture;
- Type of treatment: conservative treatment with functional appliances;
- Follow-up: > six months;
- Type of studies: prospective and retrospective human studies (in at least 10 patients), English language, with abstract available;
- Clinical/radiographical assessment: temporomandibular joint (TMJ) examination before and after treatment;
- History: absence of any systemic disease affecting bone and growth.

### 2.3. Data Extraction

The titles retrieved from the literature search were exported to an Excel spreadsheet (version 365, Microsoft Corporation, Redmond, WA, USA) and examined through a two-step procedure.

Firstly, a title/abstract screening was performed to identify which articles fulfilled the eligibility criteria for full-text evaluation. Two independent and trained reviewers (M.A.Z. and E.S.) were asked to screen the records and, for the title and abstract, decide whether it should be included or excluded from the review. Articles were selected for full-text evaluation if their title and abstract met all the inclusion criteria. If any potentially relevant article missed key information according to one of the two screeners, a senior investigator (P.G.) was consulted to attain a consensus decision. Then, full-text papers were assessed for admittance in the literature review. In the case of missing information, the authors were contacted by e-mail.

### 2.4. Quality Assessment

The three studies finally included for the qualitative analysis were all retrospective, uncontrolled, longitudinal studies. To assess the quality of the included articles, the Canada Institute of Health Economics (IHE) quality appraisal checklist and the National Institutes of Health (NIH) quality assessment tool were used, as they are specifically designed for longitudinal uncontrolled studies. The risk of bias was classified as “low risk”, “high risk”, or “unclear risk”, with the last category indicating either lack of information or uncertainty over the potential for bias [27].

## 3. Results

### 3.1. Search Results

A total of 971 articles were found from the database search. After duplicate removal, 961 potentially relevant records were identified. After the screening of titles and abstracts, eight studies were selected for full-text analysis; the main reasons for exclusion of 102 abstracts were:

- Population: about 21% of the retrieved papers (21 articles) were excluded because of the wrong age group and fracture type (studies on adults, and/or fractures of the mandibular angle or symphysis/body;
- Intervention: about 27% of the retrieved papers (27 articles) were excluded because the surgical intervention did not meet the inclusion criteria;
- Comparison: about 19% of the retrieved papers (19 articles) were excluded because they compared different plates’ and screws’ designs for rigid internal fixation of the mandible;
- Outcome: about 16% of the retrieved papers (16 articles) were excluded because they assessed only functional outcomes after conservative treatment of mandibular fractures;
- Study: about 11% of the retrieved papers (11 articles) were excluded because they were narrative or systematic reviews;
- Other reasons: about 8% of the retrieved papers (eight articles) were excluded because the full text was not available.

**Figure 1:**
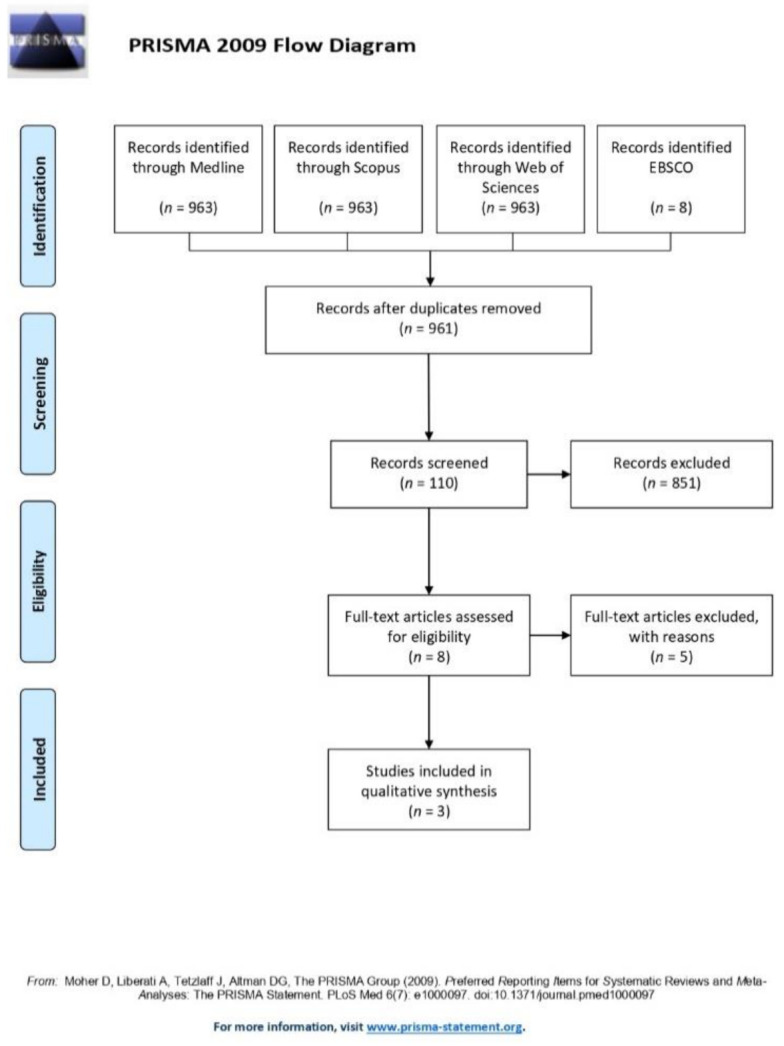
Preferred Reporting Items for Systematic Reviews and Meta-Analyses (PRISMA) flow diagram.

After full-text evaluation, five studies were further excluded due to sample size (case reports). Thus, three studies matched the eligibility criteria for the present study (Figure 1).

KahI–Nieke et al. [28] focused on radiographic parameters (mediolateral and anteroposterior widths, and condylar neck height) in patients with unilateral condylar fractures, while Liu et al. [29] and Zhao et al. [30] analyzed clinical and radiographic outcomes in patients with unilateral and bilateral condylar fractures. KahI–Nieke et al. [28] and Liu et al. [29] performed sequential computed tomographies (CT), while Zhao et al. [30] used panoramic radiographs. Due to the heterogeneity of the data, a meta-analysis could not be performed. Therefore, a qualitative summary of the findings was presented.

### 3.2. Descriptive Analysis

KahI–Nieke et al. analyzed 39 patients (3- to 16-year-olds) with unilateral condylar fractures by means of CT examinations after nine months of functional therapy [28]. The functional therapy started no later than two days after injury. The device was an occlusal splint made of acrylic resin with a height that caused 2–3 mm of mouth disclosure; it was constructed to correct and overcompensate the midline deviation, and to achieve a backward movement for patients with class I and class III occlusion, as well as 2–3 mm of mandibular advancement for a class II dental relationship. It was used for a minimum wearing time of 16 h in combination with the physiotherapy schedule (exercises to open the mouth and center the midline). The CT scans showed a slight decrease in mediolateral widths of the “healthy condyle” (−1 mm in males, −3 mm in females); on the “injured condyle”, there was a decrease of 1.9 mm in females (no difference in males) and an increase of 1 mm (both in males and females) in the mediolateral and anteroposterior widths, respectively. Moreover, the “injured condyle” showed an age-dependent shortening of the neck height, which was more pronounced when the fracture occurred to those below 10 years of age (−3.8 mm for patients < ten years vs. −1.2 mm > ten years). Fractures with and without condylar displacement were associated with a reduction of 1.6 mm of the” injured condyle” neck height, as compared to the “healthy condyle”. Nineteen patients (13.4 years old) who had received functional treatment for a unilateral condylar fracture showed at 4.9 years of follow-up a shortening of the condylar neck height, which is higher in fractures which are associated with condylar dislocation than in fractures with condylar displacement (3.6 and 1.9 mm, respectively); patients with type I fracture, according to Spiessl and Schroll (without displacement or dislocation) showed an anteroposterior difference of 1.6 mm between the affected and unaffected sides, but any difference was present at 4.9 years of follow-up. At 4.9 years of follow-up, condylar fractures with condylar displacement and dislocation showed a reduction of 1.1 mm of the anteroposterior width of the ”injured condyle”; concerning mesiodistal width, fractures with condylar displacement showed smaller changes than fractures with condylar dislocation (+0.3 and +1.3 mm, respectively).

Liu et al. analyzed 30 patients (4–8 years old) with unilateral and bilateral condylar fractures by means of clinical and radiographic examinations after three to six months of functional therapy [29]. The device was an occlusal splint made of hard acrylic resin with a height of 1–2 mm that causes mouth disclosure; no information is available about the use of a maximum laterality construction bite (contralaterally to the injured joint) for unilateral condylar fractures. The functional appliance was used full-time (except for temporary removal, such as for meals or teeth brushing); after the first month of treatment, the height of the splint was progressively reduced to keep the teeth in contact. At six months and one year after treatment, the mean unassisted interincisal opening without pain was improved from 15.8 mm (10.6–25.4 mm) before treatment (about a week after the injury) to 35.9 mm (24.7–39.8 mm) and to 38.6 mm (27.9–43.2 mm), respectively. After one year, the CT scans showed “complete” (*n* = 19) or “partial” (*n* = 11) radiological remodeling of the condylar shape and height. There was a slight increase in the mediolateral (from 15.9 mm to 16.3 mm) and anteroposterior (from 9.1 mm to 9.4 mm) widths of the “healthy condyle”; on the “injured condyle”, a decrease of mediolateral (from 16.6 mm to 16.1 mm) and anteroposterior widths (from 10.5 mm to 9.6 mm) occurred. At six months, patients showed a difference of 1.4 mm and 0.7 mm of anteroposterior and mediolateral widths between the “healthy” and “injured” condyles, but a 0.2 mm difference was present for both measurements at one year of follow-up.

Zhao et al. analyzed 40 patients (3- to 16-year-olds) with unilateral and bilateral condylar fractures by means of clinical and radiographic examinations after one to three months of functional therapy [30]. The functional therapy started three to seven days after injury. The device was an occlusal splint made of hard acrylic resin with a thickness dependent on the patient’s age, the developmental stage of the dentition, the level of the fracture, and the degree of dislocation; it was used full-time (24 hours per day) in combination with exercises of vertical opening, contralateral excursions, and protrusive movements in front of a mirror. At follow-up (14 months to 4 years), all the patients, both with unilateral and bilateral condylar fractures, showed a complete recovery of mouth opening (>35 mm), without any difference of age and gender on clinical outcomes. Only two out of 27 patients with unilateral condylar fractures showed lateral deviation >3 mm during mouth opening. On panoramic radiographs, 20 patients out of 22 with high condylar head fractures showed a 2–4 mm difference of mandibular ramus length between “healthy” and “injured” condyles, with a flattened condylar head and glenoid fossa on the “injured” side.

### 3.3. Quality Assessment

**Figure 2:**
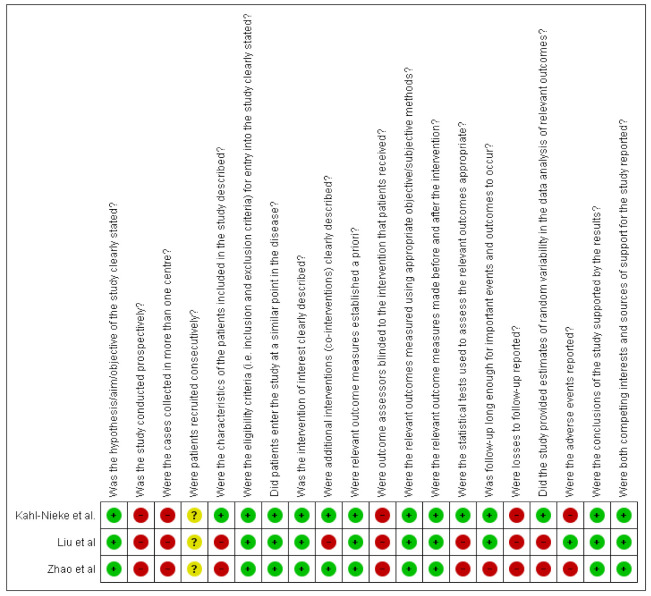
The Institute of Health Economics (IHE) quality appraisal checklist: review of authors’ judgements about each risk of bias item for each included study. Green with “+” indicates low risk, yellow with “?’” indicates unclear risk, and red with “-“indicates high risk of bias.

**Figure 3:**
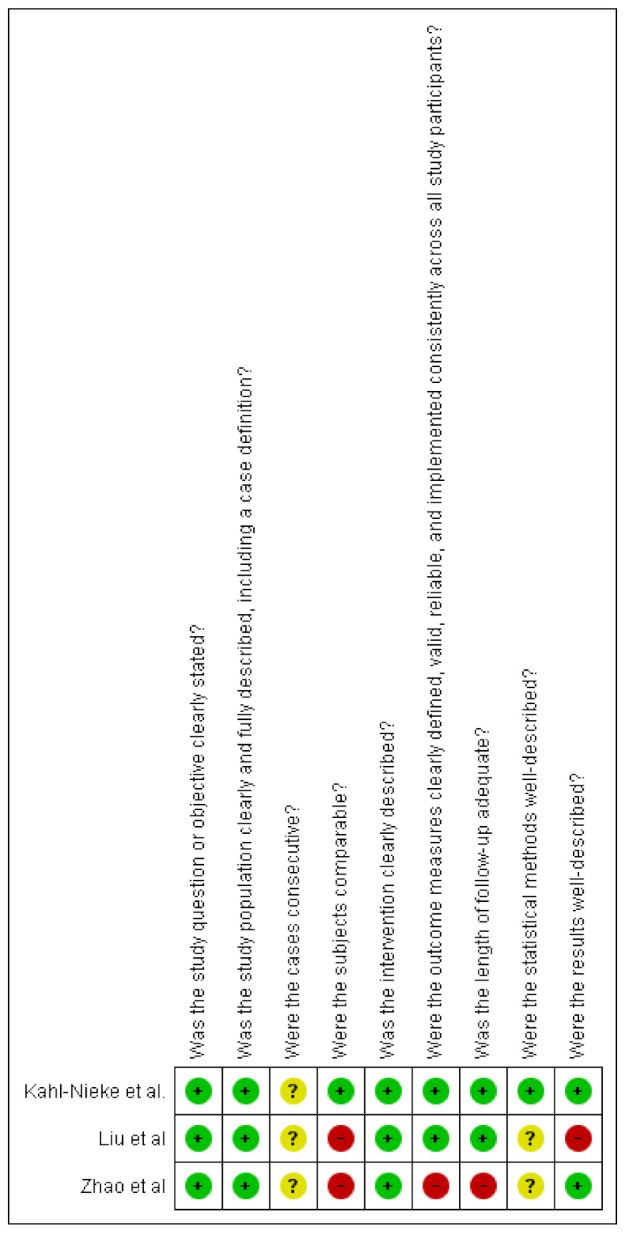
The National Institutes of Health (NIH) quality assessment tool: review of authors’ judgements about each risk of bias item for each included study. Green with “+” indicates low risk, yellow with “?’” indicates unclear risk, and red with “-“ indicates high risk of bias.

The characteristics for each included study were reported in Table 1. Kahl–Nieke et al. enrolled a homogeneous sample of patients with unilateral condylar fractures [28]; therefore, the “healthy condyle” was intended as the control group. In the other studies, unilateral and bilateral condylar fractures were included. However, Liu et al. did not analyze the type of fractures separately, while Zhao et al. made a subgroup analysis [29,30]. The “traffic light” plots tabulate the rating scores for each study in each domain; the graphs were realized with RevMan software (version 5.4.1, The Cochrane Collaboration, London, United Kingdom) (Figure 2 and Figure 3). A moderate risk of bias was detected in all the studies, due to common limitations (absence of multicenter studies, prospective design, blindness of the investigators, patients’ drop-out). An unclear risk of bias was detected for the consecutive enrolment of patients [28,29,30]. The major issues were the enrolment of unilateral and bilateral fractures, and absence of double check-measurements on X-rays. [29,30]

## 4. Discussion

The treatment with functional appliances aimed at restoring aesthetic and functional deficits of the mandible (primary defect) through joint stabilization and rehabilitation, as well as to prevent or minimize any jaw morbidity (functional disturbances, joint ankylosis) or compensatory occlusal deformity on the maxilla (secondary defect) [31].

In absence of any absolute indication for surgical treatment, a conservative approach with functional appliances allows the condylar remodeling and mandibular growth to meet the demands of function [32]. The orthopedic treatment enhances muscular function by means of disclosing teeth and increasing the vertical dimension [7,8]. The mandibular condylar cartilages are not primary sites of expansive orofacial growth, as they are not determined by craniofacial growth patterns; therefore, the mandibular macro-skeletal unit responds secondarily to the demands of their functional periosteal matrices for changes in size and shape, as well as by transformation (volumetric expansion) of the capsular matrix, and is susceptible to environmental factors [33,34].

Kahl–Nieke et al. found that, after a mean interval of 4.9 years, the difference of condylar neck length between the “injured” and the “healthy” sides was approximately 2 mm, while the variation of anteroposterior condylar width was recorded to be up to 1 mm [28]. Kahl–Nieke et al. found a higher variability of ramal height and condylar size if the fracture occurred after 10 years of age, rather than below seven. However, the absolute difference between different age subgroups fell within 0.2 mm [28]. These data are not confirmed by other studies, as Liu et al. enrolled patients aged between 4 to 8 years, while Zhao et al. included patients aged between 3 to 16 years, but did not make an age-differential analysis [29,30].

On the other hand, a fracture-related analysis revealed that fractures with condylar dislocation (Spiessl and Schroll types IV and V) are associated with twice as much condylar neck shortening (3.6 mm) as compared to the ramal length reduction (1.9 mm) of mandibular fractures with displacement (Spiessl and Schroll types II and III). Any difference of ramal height between the “injured” and “healthy” sides is evident in fractures without displacement (Spiessl and Schroll type 1) [28].

However, there was not a correlation between ramal shortening and disturbances in the occlusion:

- partial or full recovery of the joint morphology produce good to excellent functional results; Zhao et al. reported that, at one year of follow-up, all the patients (27 with unilateral, 13 with bilateral fractures) showed good to excellent clinical and functional recovery (maximum mouth opening >35 mm, lateral deviation during mouth opening <3 mm, absence of articular noise and pain, no instance of joint ankylosis, malocclusion, and facial asymmetry) [29]. Only 7% of patients with unilateral condylar fractures showed lateral deviation during mouth opening, while 8% of those with bilateral condylar fractures developed TMJ clicking. Similar results were found by Liu et al. [30]—out of 30 patients, everyone revealed good (*n* = 5) to excellent (*n* = 25) functional outcomes according to the Helkimo index [35,36].
- ramal height discrepancies may be compensated by adaptive changes, even in severe condylar displacements. Rutges et al. reported that four patients out of 28 reported a difference of more than 10 mm; in that sample, two subjects had mild, and two had moderate occlusal clinical dysfunctions [37].
- there is an absence of data collected before the injuries occurred. Rutges et al. found that the average difference of 3.4 mm of ramal height at 3.0 years’ follow-up was not significantly different from the control group, which showed a mean discrepancy of 3.0 mm between the two sides [37].

The remodeling capacity and growth potential are considerably higher if the fracture occurs before the pubertal growth spurt [38]. This finding is confirmed by the studies of Lindahl and Hollender [39,40].

**Figure 4:**
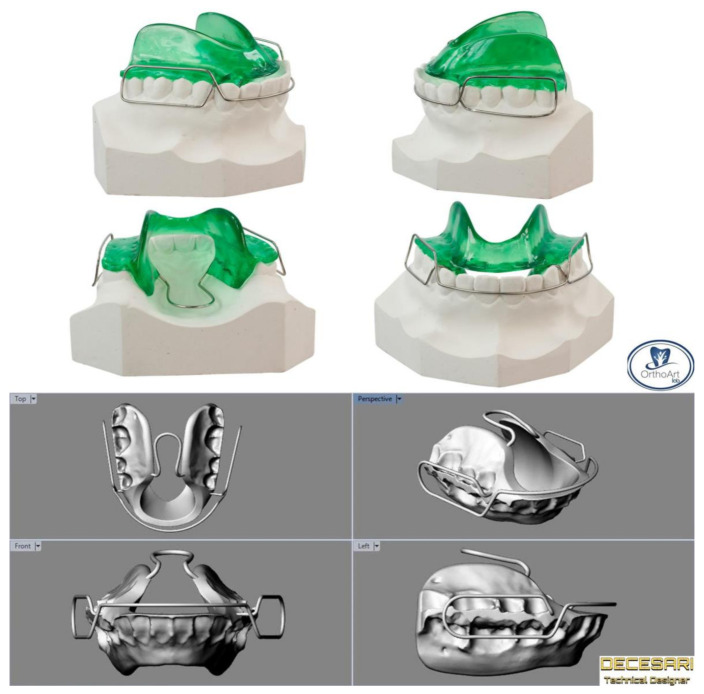
Bionator type 1 appliances realized on plaster and digital models.

Multiple appliance designs have been proposed, such as the asymmetric Bionator [8], maxillary [30], or mandibular [29] splints, with or without a midline expansion jackscrew [28], and constructed in centric occlusion, centric relation, or with a maximum laterality construction bite (contralaterally to the injured joint) [28]. All the appliances provide mouth disclosure, with a variable degree (1–3 mm) based on the age, type of fracture, and development of the dentition [28,29,30]; during periodical clinical checkups, the acrylic could be ground away from the appliance to guide dental eruption [29] (Figure 4). Conservative treatment with functional appliances lasted from 3–6 months [29] to more than one year [30]; afterwards, the activator could be used as a retainer for about 12 months, until the end of the mixed dentition phase [38].

### 4.1. Limitations

The main limitation of this study was the absence of randomized controlled clinical studies; the combined use of MeSH terms and Boolean operators aimed at increasing search sensitivity (the capacity to find any potentially relevant article). A common disadvantage of a broad search strategy is the considerable number of records from bibliographic databases that need to be screened [26]. In agreement with the Cochrane Handbook for Systematic Reviews of Interventions, non-randomized clinical trials with a moderate risk of bias were included with the aim of analyzing the current literature in terms of methodological quality and providing an in-depth insight of the research topic that could be upgraded with randomized controlled trials [41]. All the studies accounted for common risks of bias that could affect the generalizability of the results (absence of multicenter studies, prospective design, blindness of the investigators, patients’ drop-out). These limitations could be overcome by future investigations [42].

Another limitation is related to the effectiveness and predictability of conservative treatment with functional appliances, which is dependent on patients’ compliance, self-motivation, peer and authority influence, quality of life impairment and adaptability, perceived treatment progress, as well as pragmatic and recall issues, which may reduce or decrease the wearing time of the oral appliances [43,44]. Patient recommendations to improve compliance included effective communication, tailoring of prescribed wear duration, physical alteration of the appliance, and use of reminding tools [45,46].

### 4.2. Implications for Future Research

To confirm the effectiveness of functional appliances, more prospective, clinical, long-term follow-up studies with homogeneous samples of condylar fractures are deemed necessary for the incidence of postoperative wound healing problems.

Moreover, further studies could address which procedure- and patient-specific factors would yield better aesthetic, functional, and radiographic outcomes, and a lower incidence of postoperative complications.

As patient-specific factors, future studies could investigate whether the co-existence of multiple maxillary fractures, unilaterality or bilaterality, the fracture site and displacement, the state of dentition and occlusal guidance, the discal integrity, and the age/gender of the patient may affect the growth potential and remodeling capacity [47,48].

As procedure-specific factors, future studies could investigate whether appliance design, wearing time, and patients’ compliance are associated with the effectiveness of the orthopedic devices [43].

## 5. Conclusions

Even if the evidence is still questionable, short- and long-term data revealed that conservative treatment with functional appliances lead to partial or full radiological recovery of the joint morphology, along with good to excellent functional results.

## Figures and Tables

**Table 1:** Summary of the results and quality of evidence (Newcastle–Ottawa scale).

Authors	Year	Country	Type of Study	Sample	Total Score (IHE)	Total Score (NIH)
Nieke et al.	1998	Germany	Retrospective case-control	T: 19 patients C: 20 patients	14/20	8/9
Zhao et al.	2012	China	Retrospective	40 patients	11/20	5/9
Liu et al.	2013	China	Retrospective	30 patients	11/20	4/9

## Supplementary Materials

The following are available online at https://www.mdpi.com/1660-4601/17/24/9204/s1, Table S1: PRISMA Checklist. Table S2: Full electronic search strategy.
